# Association between serum phosphate levels and 28-day mortality in patients with sepsis-associated liver injury: a cohort study

**DOI:** 10.1186/s12879-025-11942-y

**Published:** 2025-11-04

**Authors:** Ting Ao, Yingxiu Huang, Peng Zhen, Ming Hu

**Affiliations:** Department of Infectious Diseases, Beijing Luhe Hospital, Capital Medical University, No.82, Xinhua South Road, Tongzhou District, Beijing, 101100 China

**Keywords:** Sepsis-associated liver injury, Phosphorus, 28-day mortality, Critical illness, Sepsis

## Abstract

**Background:**

Sepsis-associated liver injury (SALI) has a high mortality rate, but there is no established prognostic criterion for its outcomes. Although serum phosphate levels have been associated with mortality in several critical illnesses, research on their relationship with SALI is limited. This research aimed to explore the association between serum phosphate levels and 28-day mortality in SALI patients.

**Methods:**

We enrolled patients with SALI from the Medical Information Mart for Intensive Care-IV database. Serum phosphate levels were recorded within the initial 24 h of Intensive Care Unit admission. The primary outcome of interest was 28-day mortality. Multivariable Cox regression was applied to examine the association between serum phosphate and mortality. Additional subgroup analyses were carried out to ensure these findings.

**Results:**

We analyzed data from 538 SALI patients, with a mean age of 69 years, of 61.9% male. The 28-day mortality rate was 35.1%. In the unadjusted analysis, higher phosphate levels at admission were significantly linked to increased 28-day mortality (hazard ratio [HR], 1.23; 95% confidence interval [CI], 1.18 to 1.29; *p* < 0.001). After controlling for covariates such as age, sex, race, laboratory results, comorbidities, and illness severity, the association remained significant (HR, 1.13; 95% CI, 1.04 to1.22; *p* = 0.006). Subgroup confirmed the consistency of these findings.

**Conclusion:**

Higher serum phosphate levels at admission were independently linked to increased 28-day mortality in SALI patients. This suggests that serum phosphate could be a useful prognostic marker for risk stratification and clinical management in SALI.

**Clinical trial number:**

Not applicable.

**Supplementary Information:**

The online version contains supplementary material available at 10.1186/s12879-025-11942-y.

## Introduction

Sepsis is a critical state marked by organ impairment due to an inappropriate host response to infection [1]. Rudd et al. [2] reported that sepsis-related mortality is approximately 22.5%, contributing to 19.7% of global deaths. This high mortality rate imposes a significant economic burden. The liver, a central organ involved in detoxification, metabolism, and immune functions, is particularly susceptible to sepsis [3]. The development of sepsis-associated liver injury (SALI) involves a complex array of factors, including inflammatory responses, bacterial translocation, and oxidative stress [4, 5]. SALI is a crucial component of sepsis-induced multiple organ failure and is often linked with a poor prognosis [6]. Identifying effective prognostic markers is vital for the timely management of SALI.

Comprising about 1% of total body weight, phosphate ranks as the second most prevalent mineral in humans [7]. It is essential for various biological processes, including energy production, pH regulation in the blood, enzyme activation, and the function of organs such as the kidneys and immune system [8].

Several studies have demonstrated that abnormal serum phosphate levels are associated with adverse outcomes in critical illness. Hyperphosphatemia was an independent predictor of death in sepsis [9, 10]. Similar associations have been observed in patients with acute pancreatitis [11], severe burns [12], and COVID-19 [13], indicating that elevated phosphate is a consistent risk factor across diverse populations. The role of hypophosphatemia, however, remains controversial. Some studies suggested that it may serve as a marker of disease severity and is associated with an increased risk of mortality in critically ill patients [14]. Conversely, other reports described hypophosphatemia as protective, showing reduced mortality in septic patients [9, 15].

Importantly, previous studies have focused on general sepsis or critically ill populations, with no investigation specifically targeting patients with SALI, a subgroup characterized by distinct pathophysiological features and high mortality. Whether phosphate abnormalities are associated with mortality in SALI remains uncertain. To address this gap, we analyzed data from the Medical Information Mart for Intensive Care-IV (MIMIC-IV) database to examine the association between serum phosphate levels and 28-day mortality of SALI patients. Our findings provide novel insights into the prognostic significance of serum phosphate in this vulnerable group.

## Materials and methods

### Data sources and setting

Data for this investigation was sourced from the MIMIC-IV database, which provides comprehensive, de-identified electronic health records for Intensive Care Unit (ICU) admissions at Beth Israel Deaconess Medical Center (BIDMC) in Boston, MA, USA, spanning 2008–2019. Access was approved under certification number 58,844,105(First author: Ting Ao). Ethical approval was provided by the BIDMC Institutional Review Board, which waived the requirement for informed consent. The present cohort study adhered to the guidelines set forth by the Strengthening the Reporting of Observational Studies in Epidemiology (STROBE) statement [16].

### Study population

The cohort included all ICU admissions in the MIMIC-IV database who were deemed eligible. We specifically focused on adult patients meeting the Sepsis-3 definition, defined as a Sequential Organ Failure Assessment (SOFA) score increase of at least 2 points attributable to infection-induced dysregulated host response. In accordance with the Surviving Sepsis Campaign guidelines, SALI was defined as total bilirubin level greater than 2 mg/dL and an International Normalized Ratio (INR) exceeding 1.5 in sepsis patients. These criteria are broadly accepted in published studies [17–20]. The analysis was restricted to patients who had serum phosphate levels recorded during the initial 24 h following ICU admission. Patients were excluded based on the criteria listed below: (1) patients aged less than 18 years; (2) repeated hospitalizations, excluding their first ICU admission; (3) missing phosphate data; (4) pre-existing parathyroid or liver diseases prior to sepsis onset.

### Exposure

Serum phosphate levels measured within 24 h of ICU admission were used as the exposure factor. Individuals were divided into subgroups according to standard reference ranges for serum phosphate. According to the MIMIC-IV database, the reference range for normal serum phosphate levels was defined as 2.7 mg/dL to 4.5 mg/dL.

### Covariates

Using Structured Query Language (SQL), patient data from the MIMIC-IV database were extracted and systematically recorded in PostgreSQL. At the onset of sepsis, we systematically gathered initial records, which included a wide array of physiological parameters: heart rate, mean blood pressure (MBP), respiratory rate, white blood cell count (WBC), platelet count, hemoglobin, glucose, potassium, sodium, blood urea nitrogen (BUN), creatinine, bicarbonate, total calcium, phosphate, and alanine aminotransferase (ALT). Additionally, we collected patient characteristics such as age, sex, race, SOFA score, and Charlson Comorbidity Index. Information on comorbidities, including renal disease, diabetes, chronic pulmonary disease, and congestive heart failure, was ascertained via International Classification of Diseases (ICD) code.

### Outcome

The primary outcome was 28-day mortality. Secondary endpoints included in-hospital mortality, in ICU mortality, 90-day and 365-day mortality, and the number of ventilator-free, vasopressor-free, and ICU-free days during the initial 28-day period.

### Statistical analysis

As this study is retrospective, the sample size was established according to the data accessible in the database. To handle missing data, we applied nearest neighbors’ imputation. Data are expressed as frequencies (percentages) for categorical variables and as means ± SD or medians (IQR) for continuous variables. Comparisons of continuous variables were performed using either Student’s t-test or the Wilcoxon rank-sum test according to distribution, and categorical variables were analyzed using Pearson’s chi-squared.

Variables included in the analysis were chosen based on clinical relevance and established findings in the literature [15, 21]. The impact of serum phosphate levels on 28-day mortality was assessed using multivariable Cox regression models, which yielded hazard ratios (HR) and 95% confidence intervals (CI) after adjusting for key covariates. Subgroup analyses were performed to explore potential interactions with relevant covariates and to address bias, including factors such as age, sex, race, and comorbidities. Kaplan–Meier survival curves were used to evaluate 28-day mortality across different phosphate level strata, with statistical significance determined by the log-rank test. Additionally, receiver operating characteristic (ROC) curves were generated, and the area under the curve (AUC) was calculated to evaluate the predictive performance of serum phosphate levels for 28-day mortality. Finally, sensitivity analyses were conducted: (a) excluding patients with renal diseases; (b) The Modification of Diet in Renal Disease (MDRD) study equation, which incorporates age, sex, race, and serum creatinine, was used to estimate glomerular filtration rate(eGFR) [22]. Then eGFR as a continuous covariate was included in the multivariable Cox regression models.

Analyses were performed using R Statistical Software (Version 4.2.2, http://www.R-project.org, The R Foundation) and Free Statistics software (Version 2.0, Beijing FreeClinical Medical Technology CO., Ltd, Beijing, China). A *p*-value of less than 0.05 in a two-sided test was regarded as statistically significant.

## Results

### Study cohort and patients’ characteristics

**Fig. 1:**
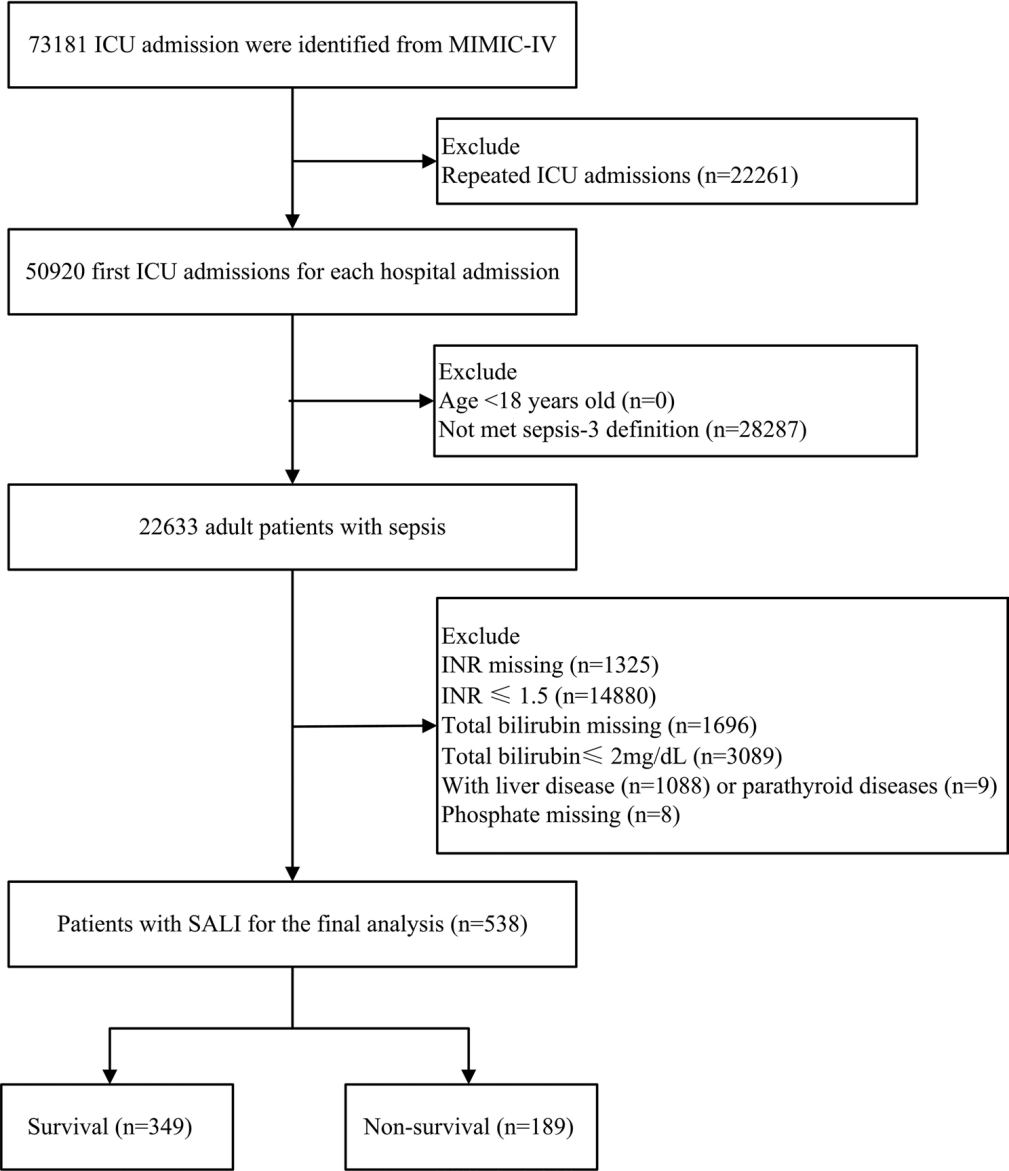
Flow chart of patient selection. Note: ICU, Intensive Care Unit; MIMIC-IV, Medical Information Mart in Intensive Care-IV; SALI, Sepsis-Associated Liver Injury; INR, International Normalized Ratio

Following stringent screening based on predetermined inclusion and exclusion criteria, a total of 538 patients were deemed eligible for inclusion in the study (Fig. 1). Among these individuals, 349 were classified as survivors (survival group) and 189 as non-survivors (non-survival group). The mean age of the study population was 69 ± 16.7 years, with males comprising 333 (61.9%) of the total cohort. In the entire cohort, those in the non-survival group had higher SOFA score and charlson comorbidity index, and more likely to have greater serum phosphate level (Table 1).

**Table 1:** Baseline characteristics of participants

Patient characteristic	Total (*n* = 538)	Survival (*n* = 349)	Non-survival (*n* = 189)	*p*
Sex, *n* (%)				0.575
Female	205 (38.1)	136 (39)	69 (36.5)	
Male	333 (61.9)	213 (61)	120 (63.5)	
Age(years), Mean ± SD	69.0 ± 16.7	67.9 ± 17.5	70.9 ± 14.8	0.050
Race, *n* (%)				0.062
White	347 (64.5)	235 (67.3)	112 (59.3)	
Other	191 (35.5)	114 (32.7)	77 (40.7)	
Vital signs				
Heart rate (bpm), Mean ± SD	93.9 ± 18.0	91.5 ± 16.8	98.3 ± 19.3	< 0.001
MBP (mmHg), Mean ± SD	74.6 ± 9.2	75.8 ± 8.7	72.6 ± 9.8	< 0.001
Respiratory rate (bpm), Mean ± SD	21.5 ± 4.4	20.9 ± 4.1	22.4 ± 4.7	< 0.001
Comorbidity disease, n (%)				
Congestive heart failure	223 (41.4)	138 (39.5)	85 (45)	0.222
Chronic pulmonary disease	120 (22.3)	80 (22.9)	40 (21.2)	0.640
Diabetes	155 (28.8)	101 (28.9)	54 (28.6)	0.928
Renal disease	120 (22.3)	71 (20.3)	49 (25.9)	0.138
Score system				
Charlson comorbidity index, Median (IQR)	6.0 (4.0, 8.0)	6.0 (4.0, 7.0)	7.0 (5.0, 9.0)	< 0.001
SOFA score, Median (IQR)	4.0 (3.0, 6.0)	4.0 (3.0, 6.0)	5.0 (4.0, 7.0)	0.004
Laboratory parameters				
Potassium (mmol/L), Mean ± SD	4.2 ± 0.9	4.1 ± 0.8	4.5 ± 1.0	< 0.001
Sodium (mmol/L), Mean ± SD	137.8 ± 5.7	137.9 ± 5.0	137.6 ± 6.8	0.527
Bicarbonate (mmol/L), Mean ± SD	19.9 ± 5.1	20.6 ± 4.9	18.5 ± 5.3	< 0.001
Hemoglobin (g/dL), Mean ± SD	10.6 ± 2.5	10.7 ± 2.5	10.2 ± 2.7	0.027
Total calcium (mg/dL), Mean ± SD	7.9 ± 1.0	8.0 ± 1.0	7.9 ± 1.1	0.270
Creatinine (mg/dL), Median (IQR)	1.4 (0.9, 2.1)	1.2 (0.9, 1.7)	1.8 (1.2, 2.7)	< 0.001
BUN (mg/dL), Median (IQR)	27.0 (17.0, 46.0)	23.0 (15.0, 35.0)	41.0 (23.0, 61.0)	< 0.001
Glucose (mg/dL), Median (IQR)	128.0 (101.0, 162.0)	126.0 (102.0, 157.0)	131.0 (98.0, 174.0)	0.408
Platelets (K/uL), Median (IQR)	146.0 (91.2, 205.8)	146.0 (100.0, 204.0)	145.0 (73.0, 209.0)	0.331
WBC(K/uL), Median (IQR)	13.5 (7.7, 20.0)	13.5 (8.5, 20.0)	13.7 (7.2, 19.9)	0.328
Phosphate (mg/dL), Median (IQR)	3.7 (2.7, 5.0)	3.4 (2.5, 4.3)	4.4 (3.5, 6.2)	< 0.001
ALT (IU/L), Median (IQR)	99.5 (34.2, 253.1)	110.0 (35.0, 232.0)	88.0 (31.0, 337.0)	0.716

Note: bpm, beats per minute; MBP, mean blood pressure; SD, standard deviation; IQR, interquartile range; SOFA, Sequential Organ Failure Assessment; BUN, blood urea nitrogen; WBC, white blood cell; ALT, alanine aminotransferase

### Association between phosphate and 28-day mortality

The overall prevalence of 28-day mortality was determined to be 35.1%. The unadjusted multivariable Cox regression analysis indicated that elevated phosphate levels upon admission were significantly linked to higher 28-day mortality. (HR, 1.23; 95% CI, 1.18 to 1.29; *p* < 0.001; Table 2). After adjusting for age, sex, race, MBP, heart rate, respiratory rate, potassium, sodium, creatinine, BUN, bicarbonate, glucose, platelets, WBC, hemoglobin, total calcium, ALT, charlson comorbidity index, SOFA, renal disease, diabetes, chronic pulmonary disease, and congestive heart failure, the association between serum phosphate and 28-day mortality remained significant. Specifically, each 1 mg/dL increment in phosphate was associated with a 13% increase in 28-day mortality risk (HR, 1.13; 95% CI, 1.04 to 1.22; *p* = 0.006).

**Table 2:** Relationship between phosphate and 28-day morality in different models

	*n*. total	*n*. event(%)	Model 1		Model 2		Model 3		Model 4		Model 5	
			HR (95%CI)	*p* value	HR (95%CI)	*p* value	HR (95%CI)	*p* value	HR (95%CI)	*p* value	HR (95%CI)	*p* value
Phosphate	538	189 (35.1)	1.23 (1.18 ~ 1.29)	< 0.001	1.24 (1.19 ~ 1.31)	< 0.001	1.19 (1.13 ~ 1.26)	< 0.001	1.12(1.03 ~ 1.21)	0.007	1.13 (1.04 ~ 1.22)	0.006

Note: HR, hazard ratio; CI, confidence interval

Model 1: unadjusted

Model 2: adjusted for sex, age, race

Model 3: adjusted for model 2 + heart rate, mean blood pressure, respiratory rate

Model 4: adjusted for model 3 + potassium, sodium, creatinine, blood urea nitrogen, bicarbonate, glucose, platelets, white blood cell count, hemoglobin, total calcium, alanine aminotransferase

Model 5: adjusted for model 4 + charlson comorbidity index, Sequential Organ Failure Assessment, congestive heart failure, chronic pulmonary disease, diabetes, renal disease

### Subgroup analyses

**Fig. 2:**
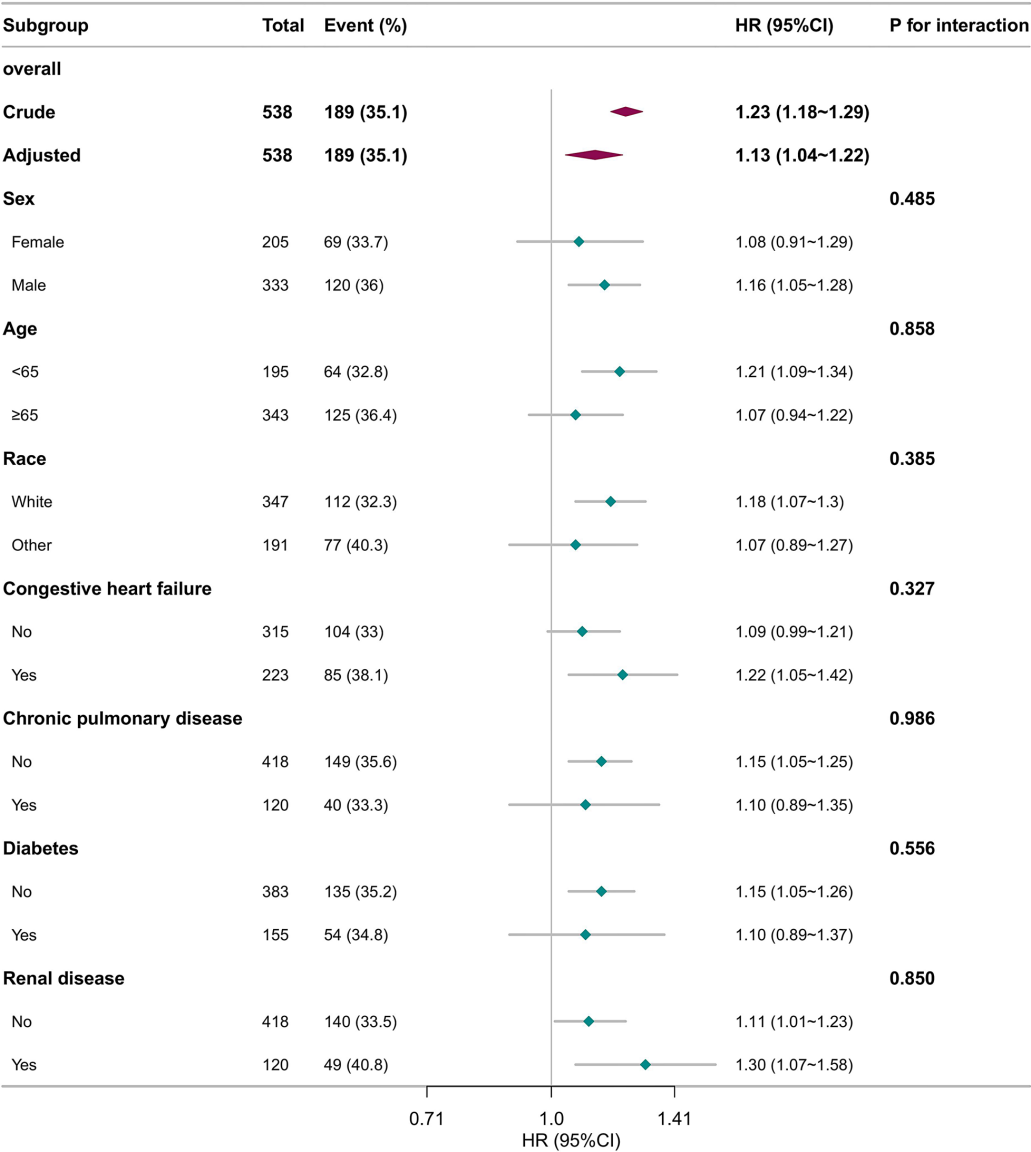
Subgroup analyses for the association of serum phosphate with 28-day mortality in SALI patients. Note: Multivariate Cox proportional hazards models were adjusted for heart rate, mean blood pressure, respiratory rate, potassium, sodium, creatinine, blood urea nitrogen, bicarbonate, glucose, platelets, white blood cell count, hemoglobin, total calcium, alanine aminotransferase, charlson comorbidity index, Sequential Organ Failure Assessment score. SALI, Sepsis-Associated Liver Injury; HR, hazard ratio; CI, confidence interval

To explore potential effect modification, subgroup analyses were undertaken based on age, sex, race, and key comorbidities, including congestive heart failure, diabetes, chronic pulmonary disease, and renal disease, examining their influence on the association between serum phosphate and 28-day mortality. Subgroup analyses revealed no statistically significant interactions (*p* > 0.05, Fig. 2), suggesting a uniform effect of serum phosphate on 28-day mortality across different groups.

### Kaplan-Meier survival curve analysis

**Fig. 3:**
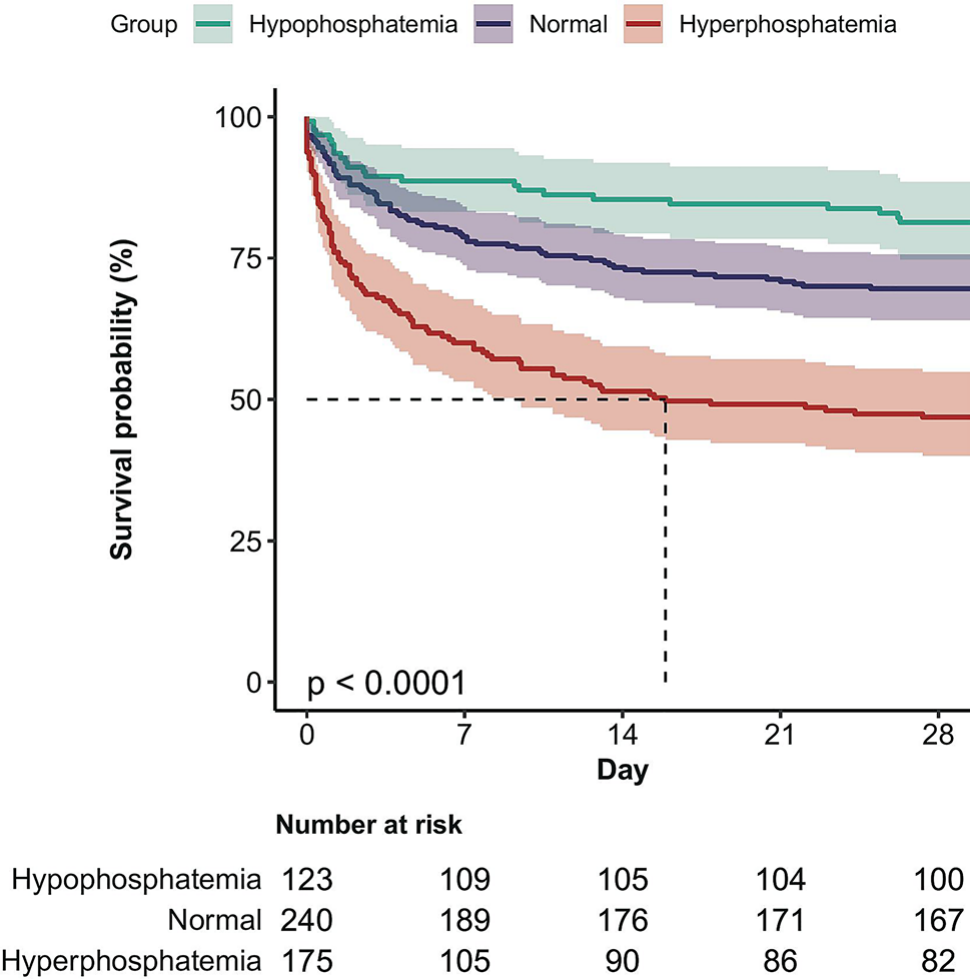
Kaplan-Meier curve for 28-day mortality according to serum phosphate

According to the normal reference ranges, we categorized phosphate into three groups, hyperphosphatemia (phosphate > 4.5 mg/dl), normal (2.7 mg/dL-4.5 mg/dL) and hypophosphatemia (phosphate ≤ 2.7 mg/dl), and performed survival curve analysis. The 28-day cumulative survival rates were found to be lower in the hyperphosphatemia group than in the other groups, as shown by the Kaplan-Meier curve (log-rank test, *p* < 0.0001) (Fig. 3).

### ROC curve analysis

**Fig. 4:**
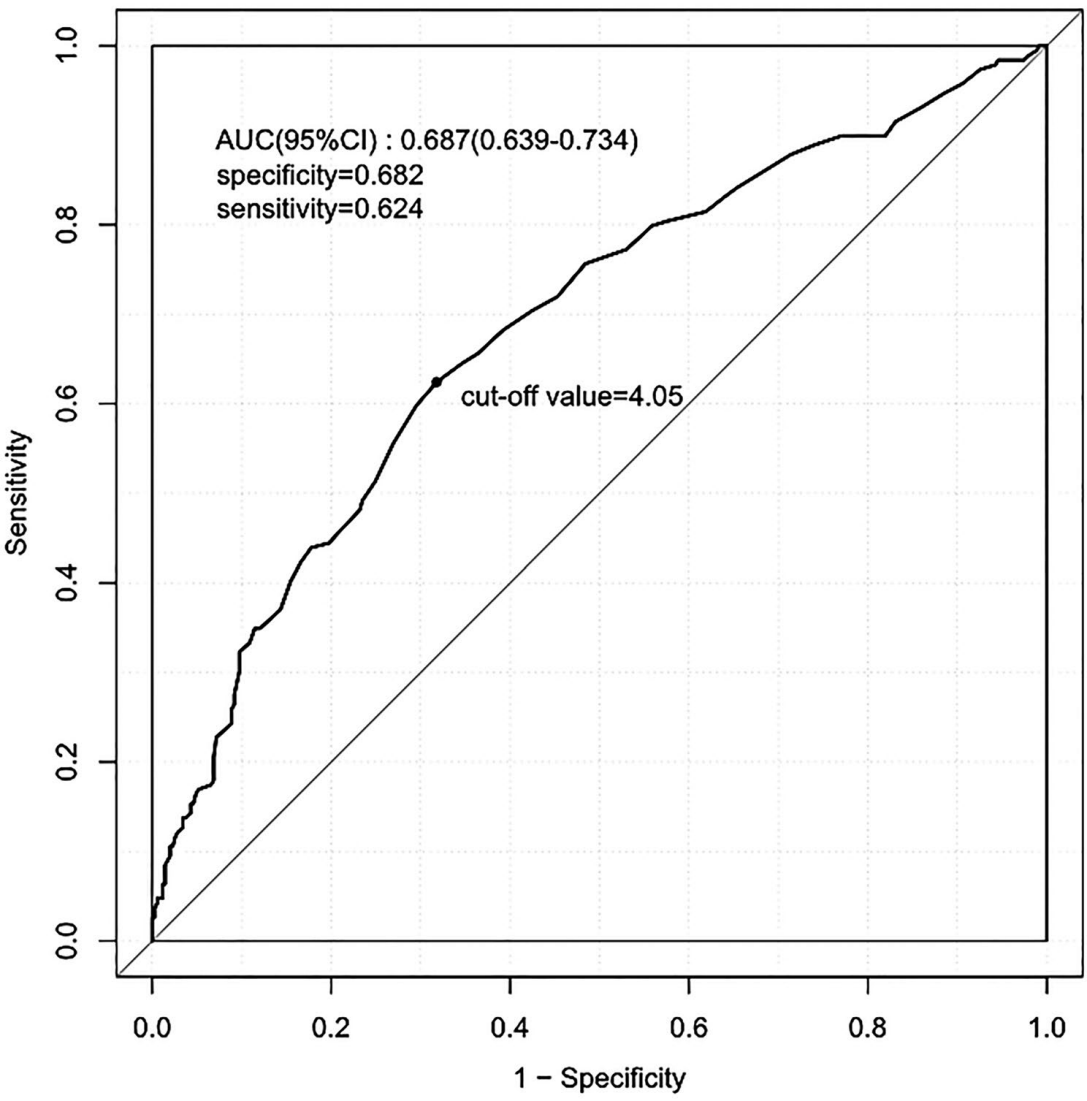
ROC curves of serum phosphate. Note: ROC, Receiver operating characteristic; AUC, Area under the curve

ROC analysis indicated that serum phosphate levels have a moderate clinical predictive value for 28-day mortality in SALI patients, with AUC of 0.687(95% CI: 0.639–0.734) (Fig. 4).

### Secondary outcomes

Higher phosphate levels were also associated with increased in-hospital mortality (HR, 1.13; 95% CI, 1.03 to 1.22; *p* = 0.006), in-ICU mortality (HR, 1.12; 95% CI, 1.03 to 1.22; *p* = 0.009), 90-day mortality (HR 1.11; 95% CI, 1.02 to 1.20; *p* = 0.017), and 365-day mortality (HR 1.11; 95% CI, 1.02 to 1.20; *p* = 0.010). For every 1 mg/dL increment in phosphate level, ICU-free days decreased by 0.95 (95% CI, -1.53 to -0.38), ventilator-free days by 1.08 (95% CI, -1.70 to -0.45), and vasopressor-free days by 1.05 (95% CI, -1.68 to -0.41) over a 28-day period (Table 3).

**Table 3:** Secondary outcome analysis

Secondary outcomes	*n*. total	*n*. event (%)	Crude model		Adjusted model	
			HR/β(95%CI)	*p*-value	HR/β(95%CI)	*p*-value
90-day mortality^a^	538	226 (42.0)	1.23 (1.17–1.28)	< 0.001	1.11 (1.02 ~ 1.20)	0.017
365-day mortality^a^	538	273 (50.7)	1.22 (1.17–1.27)	< 0.001	1.11 (1.02 ~ 1.20)	0.010
In-hospital mortality^a^	538	169 (31.4)	1.25 (1.20 ~ 1.31)	< 0.001	1.13 (1.03 ~ 1.22)	0.006
In-ICU mortality^a^	538	143 (26.6)	1.26 (1.20 ~ 1.32)	< 0.001	1.12 (1.03 ~ 1.22)	0.009
ICU-free days until 28 days^b^	538	NA	-1.87 (-2.30~-1.45)	< 0.001	-0.95 (-1.53~-0.38)	0.001
Vasopressor-free days until 28 days^b^	538	NA	-2.02 (-2.48~-1.55)	< 0.001	-1.08 (-1.70~-0.45)	0.001
Ventilator-free days until 28 days^b^	538	NA	-2.01 (-2.47~-1.54)	< 0.001	-1.05 (-1.68~-0.41)	0.001

^a^: Mortality outcomes are expressed as hazard ratios (HRs) with 95% confidence intervals (CI) derived from Cox regression models

^b^: Ventilator-free days, vasopressor-free days, and ICU-free days until 28 days were presented as β coefficients (95% CI) from linear regression models

Crude model was unadjusted

Adjusted model was adjusted for sex, age, race, heart rate, mean blood pressure, respiratory rate, potassium, sodium, creatinine, blood urea nitrogen, bicarbonate, glucose, platelets, white blood cell count, hemoglobin, total calcium, alanine aminotransferase, charlson comorbidity index, Sequential Organ Failure Assessment, congestive heart failure, chronic pulmonary disease, diabetes, renal disease

### Sensitivity analyses

After excluding patients with renal disease, the association between serum phosphate and 28-day mortality in SALI remained consistent. Following adjustment for potential confounders (excluding renal disease), a 1 mg/dL rise in phosphate was linked to a 12% increase in 28-day mortality risk (95% CI, 1.01–1.23; *P* = 0.025; Supplementary Table 1). In addition, MDRD study equation was used to calculate eGFR). Then eGFR was adjusted in the fully adjusted model. The results remained robust(HR 1.11; 95% CI, 1.02–1.21; *P* = 0.012; Supplementary Table 1).

## Discussion

Elevated serum phosphate levels were significantly associated with a greater risk of 28-day mortality in SALI patients, according to the study results. This relationship was consistently observed across various subgroup analyses. Additionally, hyperphosphatemia was associated to a heightened risk of both in-ICU and hospital mortality.

Phosphate is essential for various physiological functions, including oxygen transport, homeostasis, and cellular metabolism [10, 23]. Imbalances in serum phosphate levels, including hypophosphatemia and hyperphosphatemia, can adversely affect health and are associated with poor outcomes. Research has increasingly focused on the clinical relevance of serum phosphate. Guo et al. [10] demonstrated that every 1 mg/dL rise in serum phosphate was linked to a 19% increase in 30-day mortality risk of sepsis (95%CI,1.13 to 1.26). Elevated phosphate levels have also been linked to higher risks of hospital and ICU mortality, while hypophosphatemia was found to be protective [21]. Consistent with prior research, our study demonstrated a significant positive association between serum phosphate and 28-day mortality in patients with SALI.

However, the association between abnormal phosphate levels and outcomes in critical ill patients remains contentious. A retrospective cohort analysis found a U-shaped relationship between serum phosphate and long-term primary cardiac disease risk, with hypophosphatemia potentially increasing the risk [24]. Another research suggested hypophosphatemia was an independent risk factor for 28-day ICU mortality (adjusted odd ratio, 1.5; 95% CI, 1.1 to 2.1; *P* = 0.01) [14]. Conversely, a systematic review and meta-analysis found no significant association between pre-intervention hypophosphatemia and increased mortality risk (relative risk,0.97; 95% CI, 0.86–1.09; *P* = 0.588) [25]. Our study found that hypophosphatemia was associated with lower 28-day mortality in patients with SALI. Variations in patient cohorts, the timing of measurements, and the thresholds used to define abnormal phosphate levels could account for these differences.

The detailed mechanism through which phosphate impacts the prognosis of SALI continues to be uncertain. Excessive phosphate levels can lead to toxic effects that contribute to damage in various organs, including the liver. Phosphate has been connected to both inflammatory reactions and oxidative stress, as well as cytotoxic effects [26]. Elevated phosphate levels have been identified as an independent risk factor for inflammatory conditions and are positively associated with traditional inflammatory markers, including C-reactive protein and interleukin-6 [27]. Furthermore, oxidative stress, driven by phosphate, plays a critical role in organ injury [28, 29]. The interplay of excessive oxidative stress and inflammatory responses, combined with endothelial and epithelial damage from infection, can lead to sepsis-associated organ dysfunction [30]. The liver is particularly susceptible to this combined damage from sepsis and phosphate toxicity. Due to the retrospective nature of this observational study, the causal mechanisms underlying the observed associations cannot be determined. Additional studies are needed to explore these mechanisms and to better understand the contribution of phosphate to hepatic injury in sepsis.

Our investigation exhibits several notable advantages. Firstly, it represents one of the first systematic investigations into the link between serum phosphate levels and mortality in patients with SALI. Secondly, we accounted for a range of potential confounding factors, including renal dysfunction and parathyroid disorders. Recognizing that these conditions can markedly affect serum phosphate levels, we removed patients with parathyroid disorders and adjusted for renal conditions in our analysis. Furthermore, we controlled for other relevant variables such as vital signs, laboratory results, and comorbidities within our Cox regression models.

Despite these advantages, several limitations warrant careful consideration and cautious interpretation of our findings. Firstly, despite controlling for known confounders, the retrospective nature of our study inherently leaves room for unidentified confounders that could impact our results. Secondly, the study’s sample size might be inadequate, potentially introducing small sample bias and selection bias. Thirdly, our study design does not allow for establishing causality between serum phosphate levels and mortality. Moreover, due to the fluctuating nature of serum phosphate, a single measurement might not provide a comprehensive view of its link to mortality over time.

## Conclusion

This study demonstrated that elevated phosphate levels upon ICU admission are independently linked to a higher risk of 28-day mortality in patients with SALI. These results suggest that serum phosphate may serve as a potential prognostic marker for SALI; however, prospective studies are required to validate its clinical utility. Future research should aim to confirm these findings and investigate the mechanisms through which phosphate affects mortality in this context.

## Supplementary Information

Below is the link to the electronic supplementary material.

Supplementary Material 1

## Data Availability

The datasets presented in this study can be found in online repositories. The names of the repository/repositories and accession number(s) can be found at: https://mimic.mit.edu/.

## Abbreviations

SALI: Sepsis-associated liver injury
HR: Hazard ratio
CI: Confidence interval
MIMIC-IV: Medical Information Mart for Intensive Care-IV
STROBE: Strengthening the Reporting of Observational Studies in Epidemiology
ICU: Intensive Care Unit
BIDMC: the Beth Israel Deaconess Medical Center
SOFA: Sequential Organ Failure Assessment
INR: International Normalized Ratio
SQL: Structured Query Language
MBP: Mean blood pressure
WBC: White blood cell count
BUN: Blood urea nitrogen
ALT: Alanine aminotransferase
ICD: International Classification of Diseases
SD: Standard deviations
IQR: Interquartile ranges
ROC: Receiver operating characteristic
AUC: Area under the curve
MDRD: Modification of Diet in Renal Disease
GFR: Glomerular filtration rate

## References

[1] Singer M, Deutschman CS, Seymour CW, Shankar-Hari M, Annane D, Bauer M, et al. The third international consensus definitions for sepsis and septic shock (sepsis-3). JAMA. 2016;315(8):801–10. 26903338 10.1001/jama.2016.0287 PMC4968574

[2] Rudd KE, Johnson SC, Agesa KM, Shackelford KA, Tsoi D, Kievlan DR, et al. Global, regional, and national sepsis incidence and mortality, 1990–2017: analysis for the global burden of disease study. Lancet Lond Engl. 2020;395(10219):200–11. 10.1016/S0140-6736(19)32989-7 PMC6970225 31954465

[3] Yan M, Yu Y, Mao X, Feng J, Wang Y, Chen H, et al. Hydrogen gas inhalation attenuates sepsis-induced liver injury in a FUNDC1-dependent manner. Int Immunopharmacol. 2019;71:61–7. 30877875 10.1016/j.intimp.2019.03.021

[4] Zhang X, Liu H, Hashimoto K, Yuan S, Zhang J. The gut-liver axis in sepsis: interaction mechanisms and therapeutic potential. Crit Care Lond Engl. 2022;26(1):213. 10.1186/s13054-022-04090-1 PMC9277879 35831877

[5] Beyer D, Hoff J, Sommerfeld O, Zipprich A, Gaßler N, Press AT. The liver in sepsis: molecular mechanism of liver failure and their potential for clinical translation. Mol Med Camb Mass. 2022;28(1):84. 35907792 10.1186/s10020-022-00510-8 PMC9338540

[6] Strnad P, Tacke F, Koch A, Trautwein C. Liver - guardian, modifier and target of sepsis. Nat Rev Gastroenterol Hepatol. 2017;14(1):55–66. 27924081 10.1038/nrgastro.2016.168

[7] Calvo MS, Lamberg-Allardt CJ. Phosphorus. Adv Nutr Bethesda Md. 2015;6(6):860–2. 10.3945/an.115.008516 PMC4642415 26567206

[8] Wong SK. A review of current evidence on the relationship between phosphate metabolism and metabolic syndrome. Nutrients. 2022;14(21):4525. 36364791 10.3390/nu14214525 PMC9656201

[9] Li Z, Shen T, Han Y. Effect of serum phosphate on the prognosis of septic patients: a retrospective study based on MIMIC-IV database. Front Med. 2022;9:728887. 10.3389/fmed.2022.728887 PMC8957859 35350581

[10] Guo C, Su Y, He L, Zeng Z, Ding N. A non-linear positive relationship between serum phosphate and clinical outcomes in sepsis. Heliyon. 2022;8(12):e12619. 36619439 10.1016/j.heliyon.2022.e12619 PMC9816969

[11] Fischman M, Elias A, Klein A, Cohen Y, Levy Y, Azzam ZS, et al. The association between phosphate level at admission and early mortality in acute pancreatitis. J Gastroenterol. 2023;58(11):1157–64. 37594581 10.1007/s00535-023-02034-2

[12] Kuo G, Lee CC, Yang SY, Hsiao YC, Chuang SS, Chang SW, et al. Hyperphosphatemia is associated with high mortality in severe burns. PLoS ONE. 2018;13(1):e0190978. 29315336 10.1371/journal.pone.0190978 PMC5760089

[13] Malinowska J, Małecka-Giełdowska M, Bańkowska D, Borecka K, Ciepiela O. Hypermagnesemia and hyperphosphatemia are highly prevalent in patients with COVID-19 and increase the risk of death. Int J Infect Dis IJID Off Publ Int Soc Infect Dis. 2022;122:543–9. 10.1016/j.ijid.2022.06.057 PMC9254648 35803468

[14] Wang L, Xiao C, Chen L, Zhang X, Kou Q. Impact of hypophosphatemia on outcome of patients in intensive care unit: a retrospective cohort study. BMC Anesthesiol. 2019;19(1):86. 31122196 10.1186/s12871-019-0746-2 PMC6533764

[15] Xu X, Zhang L, Liu W, Li S, Zhao Q, Hua R, et al. Analysis of the relationship between early serum phosphate levels and short-term mortality in septic patients: a retrospective study based on mimic-iv. Shock Augusta Ga. 2023;59(6):838–45. 36947698 10.1097/SHK.0000000000002119 PMC10227928

[16] Field N, Cohen T, Struelens MJ, Palm D, Cookson B, Glynn JR, et al. Strengthening the reporting of molecular epidemiology for infectious diseases (STROME-ID): an extension of the STROBE statement. Lancet Infect Dis. 2014;14(4):341–52. 24631223 10.1016/S1473-3099(13)70324-4

[17] Yi X, Jin D, Huang S, Xie Z, Zheng M, Zhou F, et al. Association between lactate-to-albumin ratio and 28-days all-cause mortality in patients with sepsis-associated liver injury: a retrospective cohort study. BMC Infect Dis. 2024;24(1):65. 38195421 10.1186/s12879-024-08978-x PMC10775525

[18] Wen C, Zhang X, Li Y, Xiao W, Hu Q, Lei X, et al. An interpretable machine learning model for predicting 28-day mortality in patients with sepsis-associated liver injury. PLoS ONE. 2024;19(5):e0303469. 38768153 10.1371/journal.pone.0303469 PMC11104601

[19] Lei J, Zhai J, Zhang Y, Qi J, Sun C. Supervised machine learning models for predicting sepsis-associated liver injury in patients with sepsis: development and validation study based on a multicenter cohort study. J Med Internet Res. 2025;27:e66733. 40418571 10.2196/66733 PMC12149780

[20] Wang J, Hu X, Cao S, Zhao Y, Chen M, Hua T, et al. Aspirin is associated with improved 30-day mortality in patients with sepsis-associated liver injury: a retrospective cohort study based on MIMIC IV database. Front Pharmacol. 2025;16:1514392. 40103585 10.3389/fphar.2025.1514392 PMC11913821

[21] Fang Y, Zhang Y, Zhang X. Serum phosphate levels and the development of sepsis associated acute kidney injury: evidence from two independent databases. Front Med. 2024;11:1367064. 10.3389/fmed.2024.1367064 PMC10995237 38585149

[22] Levey AS, Coresh J, Greene T, Stevens LA, Zhang YL, Hendriksen S, et al. Using standardized serum creatinine values in the modification of diet in renal disease study equation for estimating glomerular filtration rate. Ann Intern Med. 2006;145(4):247–54. 16908915 10.7326/0003-4819-145-4-200608150-00004

[23] Massy ZA, Merkling T, Wagner S, Girerd N, Essig M, Wanner C, et al. Association of serum phosphate with efficacy of Statin therapy in hemodialysis patients. Clin J Am Soc Nephrol CJASN. 2022;17(4):546–54. 35236715 10.2215/CJN.12620921 PMC8993469

[24] Hayward N, McGovern A, de Lusignan S, Cole N, Hinton W, Jones S. U-shaped relationship between serum phosphate and cardiovascular risk: a retrospective cohort study. PLoS ONE. 2017;12(11):e0184774. 29117214 10.1371/journal.pone.0184774 PMC5695582

[25] Wei S, Li Y, Zhang C, Guo X, Liang X, Huang Y, et al. Prognostic value of serum phosphate levels in sepsis: a systematic review and meta-analysis. PeerJ. 2023;11:e16241. 37849826 10.7717/peerj.16241 PMC10578301

[26] Michigami T, Yamazaki M, Razzaque MS. Extracellular phosphate, inflammation and cytotoxicity. Adv Exp Med Biol. 2022;1362:15–25. 35288869 10.1007/978-3-030-91623-7_3

[27] Navarro-González JF, Mora-Fernández C, Muros M, Herrera H, García J. Mineral metabolism and inflammation in chronic kidney disease patients: a cross-sectional study. Clin J Am Soc Nephrol CJASN. 2009;4(10):1646–54. 19808245 10.2215/CJN.02420409 PMC2758261

[28] Chung LH, Liu ST, Huang SM, Salter DM, Lee HS, Hsu YJ. High phosphate induces skeletal muscle atrophy and suppresses myogenic differentiation by increasing oxidative stress and activating Nrf2 signaling. Aging. 2020;12(21):21446–68. 33136552 10.18632/aging.103896 PMC7695395

[29] Hsu YJ, Chang GJ, Lai YJ, Chan YH, Chen WJ, Kuo CT, et al. High-phosphate diet causes atrial remodeling and increases atrial fibrillation vulnerability via STAT3/NF-κB signaling and oxidative stress. Acta Physiol Oxf Engl. 2023;238(2):e13964. 10.1111/apha.13964 36929808

[30] Zhang W, Jiang H, Wu G, Huang P, Wang H, An H, et al. The pathogenesis and potential therapeutic targets in sepsis. MedComm. 2023;4(6):e418. 38020710 10.1002/mco2.418 PMC10661353

