# Supplementary material for: Association between serum phosphate levels and 28-day mortality in patients with sepsis-associated liver injury: a cohort study
Source: BMC Infect Dis. 2025 Nov 4;25:1494. doi: 10.1186/s12879-025-11942-y (PMC12584543; doi:10.1186/s12879-025-11942-y)
Supplement: Supplementary file 1 — Supplementary Material 1 [file 12879_2025_11942_MOESM1_ESM.docx]

Supplementary table 1 Sensitivity Analyses

| Analysis | Total | Event (%) | Crude model | | Adjusted model | |
| --- | --- | --- | --- | --- | --- | --- |
|  |  |  | HR (95%CI) | *p*-value | HR (95%CI) | *p*-value |
| Excluding patients with renal diseases^a^ | | | | | | |
| Phosphate | 418 | 140 (33.5) | 1.22 (1.15~1.28) | <0.001 | 1.12 (1.01~1.23) | 0.025 |
| Adjusted for eGFR in the fully adjusted model^b^ | | | | | | |
| Phosphate | 538 | 189 (35.1) | 1.23 (1.18~1.29) | <0.001 | 1.11 (1.02~1.21) | 0.012 |

^a^: Adjusted model was adjusted for sex, age, race, heart rate, mean blood pressure, respiratory rate, potassium, sodium, creatinine, blood urea nitrogen, bicarbonate, glucose, platelets, white blood cell count, hemoglobin, total calcium, alanine aminotransferase, charlson comorbidity index, Sequential Organ Failure Assessment, congestive heart failure, chronic pulmonary disease, diabetes

^b^: Adjusted model was adjusted for sex, age, race, heart rate, mean blood pressure, respiratory rate, potassium, sodium, creatinine, blood urea nitrogen, bicarbonate, glucose, platelets, white blood cell count, hemoglobin, total calcium, alanine aminotransferase, charlson comorbidity index, Sequential Organ Failure Assessment, congestive heart failure, chronic pulmonary disease, diabetes, renal disease, eGFR

Note: HR, hazard ratio; CI, confidence interval
